# Individual Differences in Risky Decision-Making Among Seniors Reflect Increased Reward Sensitivity

**DOI:** 10.3389/fnins.2012.00111

**Published:** 2012-07-17

**Authors:** James F. Cavanagh, David Neville, Michael X Cohen, Irene Van de Vijver, Helga Harsay, Poppy Watson, Jessika I. Buitenweg, K. Richard Ridderinkhof

**Affiliations:** ^1^Department of Cognitive, Linguistic and Psychological Sciences, Brown UniversityProvidence, RI, USA; ^2^Department of Psychology, Amsterdam Center for the Study of Adaptive Control in Brain and Behavior, University of AmsterdamAmsterdam, Netherlands; ^3^Department of Physiology, University of ArizonaTucson, AZ, USA; ^4^Cognitive Science Center Amsterdam, University of AmsterdamAmsterdam, Netherlands

**Keywords:** aging, BART, impulsivity, cognitive modeling, reward

## Abstract

Increasing age is associated with subtle but meaningful changes in decision-making. It is unknown, however, to what degree these psychological changes are reflective of age-related changes in decision quality. Here, we investigated the effect of age on latent cognitive processes associated with risky decision-making on the Balloon Analog Risk Task (BART). In the BART, participants repetitively inflate a balloon in order to increase potential reward. At any point, participants can decide to cash-out to harvest the reward, or they can continue, risking a balloon pop that erases all earnings. We found that among seniors, increasing age was associated with greater reward-related risk taking when the balloon has a higher probability of popping (i.e., a “high risk” condition). Cognitive modeling results from hierarchical Bayesian estimation suggested that performance differences were due to increased reward sensitivity in high risk conditions in seniors.

## Introduction

The trajectory of cognitive change associated with aging suggests that some presumably stable cognitive traits can actually vary across the lifespan. The prevalence and predictability of these late life changes suggest that common underlying factors may contribute to these effects, yet it is difficult to identify and differentiate such latent cognitive constructs. A recent meta-analysis has suggested that age-related change in decision quality varies when learning is involved (Mata et al., 2011). When task dynamics are explicitly understood, differing decision strategies between age groups are less likely. In contrast, more ambiguous circumstances are characterized by potentially maladaptive decisions in seniors – specifically in risky situations. Here, we investigated the effect of age on risky decision-making as assessed by the Balloon Analog Risk Task (BART; Lejuez et al., 2002). We sought to determine what cognitive factors specifically contribute to performance differences using computational methods that facilitate an understanding of complex performance patterns. Cognitive modeling offers a promising method for objectively uncovering such latent parameters (Busemeyer and Stout, 2002), which may provide closer reflections of the unobservable computations that contribute to observable behavior (Yechiam et al., 2005; O’Doherty et al., 2007).

In the BART, participants repetitively inflate or pump a balloon in order to increase potential reward. At any point, participants can decide to cash-out to harvest the reward, or they can continue, risking a balloon pop that erases all earnings. The BART has good reliability (White et al., 2008) and generalizability to real life impulsive behaviors, as demonstrated by correlations between BART pumps/pops and self-reported psychopathy, sensation seeking, impulsivity, drug and alcohol use, gambling, and unprotected sex (Lejuez et al., 2003; Hunt et al., 2005; Wallsten et al., 2005). Previous findings have detailed how seniors are characterized by risk aversive behavior on the BART (Henninger et al., 2010; Rolison et al., 2011). Intriguingly, these findings stand in contrast to results from the meta-analysis that found that seniors were usually characterized by risk-seeking behavior when optimal performance had to be learned (Mata et al., 2011). While BART performance is clear to interpret, it is difficult to determine what motivates different performance styles, especially when learning is involved. For example, an increased number of pumps could reflect impulsive risky decision-making, yet it could also reflect a more optimal decision strategy since participants often overestimate risk (Lejuez et al., 2002; Rao et al., 2008). Moreover, either of these motivations could be orthogonal to accurate learning.

In light of recent findings that age-related deficits in executive control may be unspecific and inaccurate (Verhaeghen, 2011), cognitive process models offer an opportunity to parse variance in performance to relevant latent constructs related to decision-making. For example, cognitive modeling has revealed how age-related variability in response times may be due to a benign impact of generalized slowing, not task-specific manipulations that purportedly measure executive control (Ratcliff et al., 2006). More germane to the current investigation, Wood et al. (2005) have demonstrated that in the Iowa gambling task, young adults integrate reward expectations across a prolonged trial history, whereas seniors focus more on the most recent trials and are therefore more sensitive to incidental violations of probabilistic contingencies. Such cognitive process models offer a method for identifying candidate underlying processes that occur behind the scenes of observable behavior.

Capitalizing on a previously published cognitive model of BART performance (Wallsten et al., 2005; van Ravenzwaaij et al., 2011), here we aim to decompose observable behaviors to latent components reflecting reward sensitivity, behavioral consistency, and learning-related estimation of task probabilities. These latent parameters can offer a more specific explanation of the cognitive processes underlying observable behaviors.

## Materials and Methods

### Participants

Young participants (aged 18–30) were recruited from the University of Amsterdam campus. Senior participants (above age 60) were recruited from the SeniorLab database (www.seniorlab.nl) of healthy self-selected older adults. Subjects received course credits or financial compensation for participation. They gave written informed consent before experimentation. All procedures were executed in compliance with relevant laws and institutional guidelines and were approved by the local ethics committee. The demographics of the final participant groups are as follows. Young participants: *N* = 23, female = 12, mean age = 21, age range = 18–26; senior participants: *N* = 29, female = 22, mean age = 73, age range = 63–87. Young and senior participants did not differ in their verbal intelligence, as assessed with the Nederlandse Leesvaardigheidstest voor Volwassenen (Dutch Reading test for Adults; Schmand et al., 1991) or in their working memory, as assessed with the O-span (Turner and Engle, 1989) scored using the partial-credit unit scoring system (Conway et al., 2005), *t*s < 1.5, *p*s < 15.

### Balloon analog risk task

**Figure 1:**
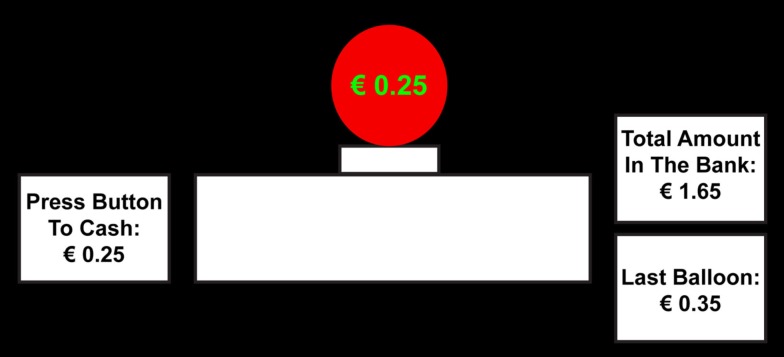
**Balloon Analog Risk Task (BART)**. Participants inflated a balloon until they decided to cash-out or until it popped and the accrued earnings on that trial were lost. Participants completed both high and low risk blocks of the BART.

Participants performed an adjusted version of the BART. A red balloon was presented in the center of a computer screen (see Figure 1). Participants could inflate the balloon by pressing the space bar, or cash the current virtual value of the balloon into a virtual bank by pressing the right shift button. On every pump the balloon could also explode; the probability of explosion was varied in two different risk conditions described below. If a balloon popped, the value of that balloon was lost to the participant, but the total amount that was previously cashed to the virtual bank was unaffected. The current value of the balloon was presented on the balloon in green digits, and in a separate box on the left side of the balloon. Two boxes on the right side indicated how much virtual money was earned with the previous-balloon, and how much virtual money the participant had collected on the virtual bank.

The starting radius of the balloon was 150 pixels; the starting value was € 0.00. On every pump the radius of the balloon increased with 1.2 pixels and the value of the balloon with € 0.05. The adjustment of the size and current value (on the balloon and in the left box) was accompanied by the sound of air entering the balloon for 100 ms. On every pop a picture of an exploded balloon was presented for 1000 ms, accompanied by the sound of an explosion. The current value of the balloon and the value in the previous-balloon box were set to € 0.00 and a new balloon was presented. On every cash moment a yellow dollar sign was presented in the middle of the screen for 1000 ms, accompanied by the sound of an old cash register. The amount of money in the bank box and the previous-balloon box were adjusted to include the current earning, the current value of the balloon was set to € 0.00, and a new balloon was presented. The response window for the participant was unlimited.

Participants first received two short blocks of training. The first block consisted of five balloons. In this block participants could only pump the balloon until it popped; they could not cash-out yet. The five balloons would pop on the 7th, 18th, 28th, 42nd, and 56th pumps (in random order). The second practice block consisted of 10 balloons and was similar to the real task, but with an explosion probability of 3.75% (average of the two real blocks). In the test phase, participants were presented with two blocks of 40 balloons. The chance that the balloon would explode was 2.5 and 5% within each block. Although this constant probability is different than the increasing probability schedule used in most studies of the BART, this manipulation allowed us to explicitly assess performance during low (2.5%) and high (5%) risk conditions.

The order of the blocks was randomized between participants. Participants were informed about the risk prior to beginning each block, and they were instructed to try to maximize the amount of virtual money in the bank. To encourage this, 5% of the virtual money in the bank was paid to the participants in addition to the payment for participation. Outcome variables for each block included the average number of pumps on cash trials, the number of popped balloons, as well as the amount of virtual money earned at the end of the block. In addition, the ratio of the number of pumps following a cash-out to the number of pumps following a pop was included as a measure of reward-based risk taking. The evolution of performance across time was investigated by splitting each block of 40 trials into four bins of 10 trials each. This analysis was performed to investigate if age-related differences were specific to early trials (as in Rolison et al., 2011), which might suggest differences in initial learning about task contingencies.

### Computational modeling

To decompose observable behavior into separable latent elements we used a hierarchical Bayesian extension of the best-fitting Wallsten et al. (2005) model, as detailed by van Ravenzwaaij et al. (2011). In hierarchical modeling, individual participants are nested within group (young and senior) and condition (low and high risk) categories, facilitating simultaneous parameter estimation for each condition for each participant. Wallsten et al. (2005) tested a variety of cognitive process models to distil latent factors influencing BART task performance. The best-fitting model (#3 in Wallsten et al., 2005) assumed that the decision maker updates the probability of explosion after each balloon, and slowly learns to estimate the explosion probability. Four free parameters were fit that describe variability between decision-making styles: two learning-related parameters alpha (α) and mu (μ), a reward sensitivity parameter gamma (γ^+^), and a behavioral consistency parameter beta (β). These parameters are referred to with Greek letters for the description of computational algorithms; text will be used otherwise.

The model for each decision maker begins with the assumption that the probability of the balloon bursting on each trial *k* is constant: $p_{k}^{\text{belief}}.$ This means that the balloon is equally likely to explode on the first pump as in the fourth, for example. The first trial starts with an *a priori* belief of the probability of explosion captured by a beta distribution with free scaling parameters α_0_ and μ_0_. This prior belief is then updated according to Bayes’ rule to calculate an updated belief of bursting. The probability of explosion for the balloon on any given trial is:

$$p_{k}^{\text{belief}}=1-\frac{\alpha_{0}+\overset{k-1}{\underset{K=0}{\sum }}n_{K}^{\text{success}}}{\mu_{0}+\overset{k-1}{\underset{K}{\sum }}n_{K}^{\text{pumps}}}\;\text{with}\;\alpha <\mu$$

The prior belief is represented by the ratio 1 − α_0_/μ_0_. This value then is updated by adding for the numerator the sum of all successful pumps so far (excluding the current trial), $\sum_{K=0}^{k-1}n_{K}^{\text{success}},$ and for the denominator by adding the sum of all pumps so far, $\sum_{K}^{k-1}n_{K}^{\text{pumps}}.$

The next component in the model specifies the number of pumps considered optimal. The free parameter γ^+^ influences the assessment of the optimal number of pumps by weighting the estimated belief of a pop $p_{k}^{\text{belief}}.$ Note that larger γ^+^ values leads to more pumps. Notably, larger estimated γ^+^ parameters have been correlated with a greater propensity for real world risky behaviors, including drug use, unprotected sex, and stealing as reported in Wallsten et al. (2005). For trial *k* the optimal number of pumps ω*_k_*, is as follows:

$$\omega_{k}=\frac{-y^{+}}{\ln\left(1-p_{k}^{\text{belief}}\right)}\;\text{with}\;y^{+}\geq 0$$

The actual probability of pumping the balloon on any opportunity *l* for trial *k* depends on both the optimal number of pumps ω*_k_*, and on the free parameter β which reflects behavioral consistency. A larger β parameter reflects a sharper, more deterministic response strategy. For example, if β = 0, then the $p_{\text{kl}}^{\text{pump}}=0.5$ and the decision maker will choose randomly between pumping and cashing. As β increases, behavior becomes more and more deterministic as defined by the optimal number of pumps (*l *− ω*_k_*). A logistic equation is used to estimate response choices with free parameter β:

$$p_{\text{kl}}^{\text{pump}}=\frac{1}{1+{\text{e}}^{\beta \left(l-\omega_{k}\right)}}\;\text{with}\;\beta \geq 0$$

In the context of Bayesian statistics, van Ravenzwaaij et al. (2011) extended previous modeling work on the BART task by introducing a hierarchical extension for the BART models. The Bayesian approach combined with hierarchical modeling has several advantages over standard approaches (i.e., maximum likelihood estimation), primarily by providing more precise parametric estimates while simultaneously estimating both subject and group level effects (Wagenmakers et al., 2008; Wetzels et al., 2010; Lee, 2011).

The hierarchical extension draws individual parameters γ^+^ and β from normal distributions around estimated group level parameters γ^+^* and β*. The learning parameters α and μ were kept as subject level parameters since they present a high degree of correlation and do not significantly affect the precision of γ^+^ and β estimates (van Ravenzwaaij et al., 2011). Whereas a standard maximum likelihood approach to model estimation would estimate parameter values which maximize the (log) likelihood of the model predicting the data, Bayesian models instead follow a different approach. Each model parameter is estimated by a probability function with a unique mean and variance. These functions are initially set to a uniform or uninformative distribution and are updated with experience according to Bayes’ rule.

A suitable numerical routine to sample from the posterior distributions is offered by the Gibbs Sampling algorithm and Markov Chain Monte Carlo (MCMC) simulations. MCMC relies on simulating one or more chains of random values sampled from the posterior distribution until all the chains have converged. Once convergence has been reached, successive samples can be assumed to be drawn from the posterior distribution representing the belief of the decision maker after experience. It is common procedure to discard initial samples (burn-in) to assure independence of the final samples from the starting chain values.

In all of the reported simulations the estimates are based on 15,000 iterations after 10,000 iterations of burn-in. For the parameters α and μ, uniform distributions were used as uninformative priors. For the parameters γ^+^ and β, Gaussian distributions centered on their group level means, γ^+^* and β* were used. For these group level parameters γ^+^* and β*, uniform distribution were once again used as uninformative priors. Three chains were used in all simulations with random starting values. MCMC sampling was implemented via the open package OpenBugs (Lunn et al., 2009) interfaced through the statistical program R. Chain convergence was assessed by means of the Rhat statistic, a scaling factor which approaches a value of 1 under chain convergence.

Note that the size of the learning rate parameters alpha and mu were scaled to the specific task used here (due to the constant probability of explosion) and thus are different from previous studies that used a larger range (c.f. Rolison et al., 2011). To facilitate comparison across studies, probability density functions were computed based on the alpha and mu parameters. The mean and variance of these functions were computed for each participant in each risk condition; these variables were then compared to examine potential differences in learning that would affect beliefs in the chance the balloon would not pop (the prior).

To summarize, like van Ravenzwaaij et al. (2011), we used a hierarchical estimation procedure to estimate gamma (γ^+^) and beta (β) parameters in the best-fitting Wallsten et al. (2005) model. However, the two learning rate parameters alpha (α) and mu (μ) remained subject level, since hierarchical estimates provided worse fits to the data (i.e., there did not appear to be any group level regularities in these variables). Therefore our model assumed group level variance for risk sensitivity and behavioral consistency, and individual differences only for learning through experience. This hybrid hierarchical procedure created the best model given that: (1) it fit better than a baseline model which assumed no learning (Wallsten et al., 2005) as assessed by Deviance Information Criteria (all DIC learning < baseline, learning mean = 137, baseline mean = 179), (2) it had full convergence where only gamma and beta parameters from the fully hierarchical van Ravenzwaaij et al. (2011) model converged, and (3) the hierarchical fits improved on the single-subject level estimation used by Wallsten et al. (2005).

## Results

### Performance

**Figure 2:**
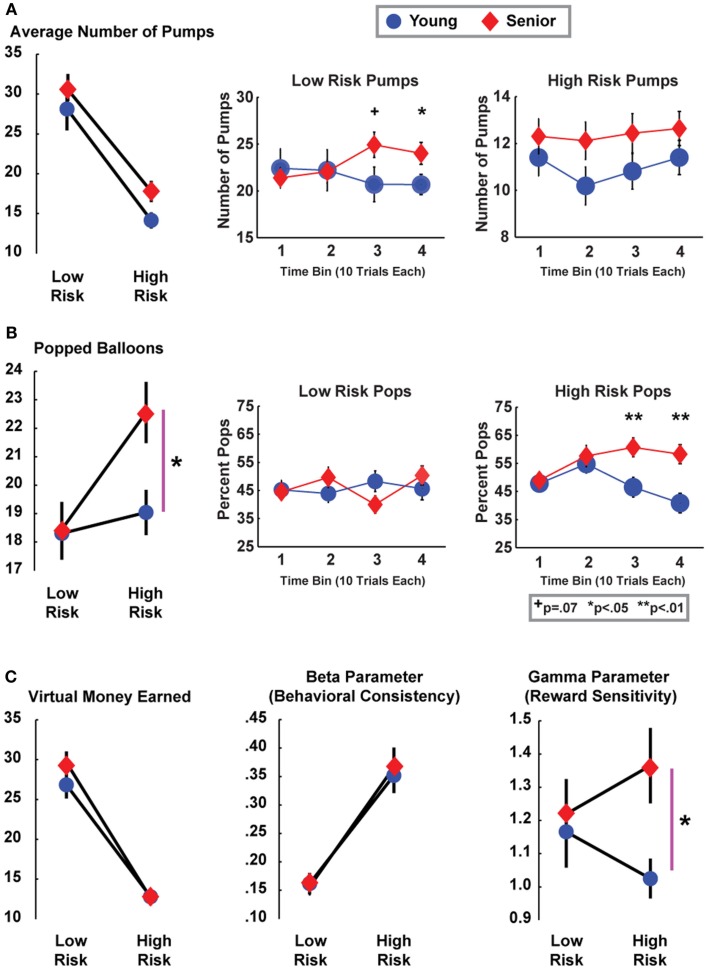
**Performance means (SEM) for BART outcomes and model parameters**. **(A)** Although the average number of pumps was nominally larger in seniors, age groups did not significantly differ from each other with the exception of late trials in the low risk condition. **(B)** Age groups significantly differed in the average number of popped balloons in the high risk condition, particularly later in the block. **(C)** The decision quality underlying these patterns is difficult to interpret, since groups did not differ in the amount of virtual earnings. However, cognitive modeling revealed that these performance differences were associated with a heightened reward sensitivity parameter in seniors in the high risk condition.

Given that the focus of this paper is on aging, only main or interactive effects with age are described. There were no main or interaction effects for age group on the average number of pumps on cash-out trials (*F*s < 1). However, there was a significant interaction between risk and age group for number of popped balloons [*F*(1, 50) = 4.56, *p* = 0.038], without a main effect of age [*F*(1, 50) = 2.21, *p* = 0.14], see Figure 2. Contrasts revealed that seniors differed in the number of pops between high and low risk conditions (*p* < 0.01) and age groups differed in the high risk condition (*p* = 0.015). To investigate how performance changed over time, data from each condition was split into four consecutive 10-block bins. In the low risk condition, there was a significant interaction between age group and time [*F*(3, 50) = 3.80, *p* = 0.02], where seniors had more pumps in the third (*p* = 0.07) and fourth (*p* = 0.05) blocks. In the high risk condition, there was a significant interaction between age group and time [*F*(3, 50) = 4.087, *p* < 0.01], where contrasts revealed that seniors had more pumps in the last two blocks specifically (*p*s < 0.01). These performance differences occurred in the absence of a difference in the amount of virtual money earned (*F*s < 1.9).

**Figure 3:**
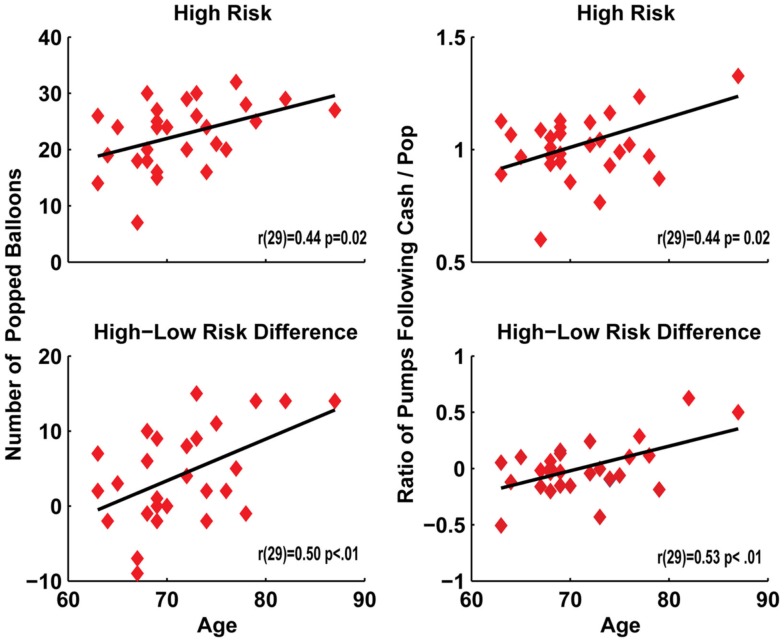
**Age correlated with the number of popped balloons and post-cash: post-pop ratio of the number of pumps in both the high risk condition, as well as the increase between the high and low risk conditions**.

Figure 3 demonstrates how in the senior group, age was correlated with the number of pops in the high risk condition [*r*(29) = 0.44, *p* = 0.02] and with the difference in pops between high and low risk conditions [*r*(29) = 0.50, *p* < 0.01]. Age also correlated with an increased ratio of the number of pumps after a cash-out compared to number of pumps after a pop in the high risk condition [*r*(29) = 0.44, *p* = 0.02] and with the difference in ratios between the high and low risk conditions [*r*(29) = 0.53, *p* < 0.01]. However, after removal of the largest outlying value only this difference measure remained significant (high risk *p* = 0.09). This integrated set of findings suggests that increasing age is associated with greater reward-based risk taking.

### Model results

The cognitive model fit four parameters for each participant during each condition: learning rate parameters alpha and mu, reward sensitivity parameter gamma, and behavioral consistency parameter beta. The reported parameter estimates were all drawn after convergence of chains (Rhat = 1). The young and senior groups were drawn from the same posterior distributions in low and high risk conditions. This suggests that the individual estimates of the probability of non-explosion (calculated as a beta distribution from the alpha and mu parameters) would be the same between age groups for each condition. When comparing the means of the distributions there was a significant effect of risk, with the high risk condition having lower estimated probabilities of non-explosion [*F*(1, 50) = 40.30, *p* < 0.01], yet there were no main or interactive effects with group, and no effects for the variance of the distributions.

To check whether learning changed over the course of the hard condition, the means of these distributions for each of the four blocks of 10 trials were estimated. There were no significant differences between age groups in the estimated probability of non-explosion over time (all *p*s > 0.11). In fact, the young group actually had marginally higher estimates of non-explosion in three out of four bins, providing evidence that differential performance in seniors was not due to a more optimistic learned belief. In conjunction with the finding of increased pops late in the high risk condition, these null effects suggest that the age groups were not characterized by differential learning of explosion probabilities during task performance.

There were no main or interactive effects with age for the beta (behavioral consistency) parameter (*F*s < 1). However, there was a significant interaction between risk and age group for the gamma (reward sensitivity) parameter [*F*(1, 50) = 4.79, *p* = 0.033], with no main effects (Figure 2C). The only significant contrast was between age groups in the high risk condition, where seniors were characterized by greater reward sensitivity [*t*(51) = 2.46, *p* = 0.017]. Condition-specific gamma parameters did not significantly correlate with age, pumps, pops, virtual earnings, or the post-cash:post-pop ratio in either age group. Thus, this model parameter reflects a distinct measure of reward-related decision-making.

## Discussion

This investigation revealed that increased age was associated with altered BART performance reflective of greater reward sensitivity during high risk decisions. In fact, age directly correlated with both pops and post-reward risk taking in the high risk condition without an increase in earnings. This performance style did not appear to depend on differences in learning: seniors had greater high risk reward sensitivity in the context of similar estimation of reward probabilities.

### Age-related alteration of performance

Seniors did not differ from the young group in the overall amount of virtual money earned in either risk condition, and the number of pumps on cash-out trials was not significantly different from the young group. While seniors were characterized by a greater number of popped balloons than young adults, and increasing age amongst seniors predicted a greater number of pops, it is difficult to determine whether these performance features reflect a behavioral indicator of poor decision-making. In short, it is difficult to know if seniors were suboptimal impulsive performers or if they were simply following a different strategy – there was no ultimate difference in earnings between the groups.

The review of Mata et al. (2011) concluded that age-related decision changes appear to be due to impairments in learning. These age-related changes were most often associated with an increase in risk-seeking behaviors, with the specific exception of two previous findings from the BART. These studies described how seniors were more risk averse (fewer pumps) when tested with only ten trials (Henninger et al., 2010; Rolison et al., 2011), yet behavioral trends converged with younger participants over a greater number of trials (Rolison et al., 2011). As shown in Figure 2, age-related differences in this investigation did not appear to be due to early task performance or learning. Rather, the age-related differences reported here were most prevalent late in each task.

The probabilistic task structure used here may have contributed to different findings between the current investigation and two previous investigations. As opposed to other studies of the BART, the probability of a pop in the current investigation did not increase with each pump; rather it remained a constant value over time. While this modification facilitated the comparison of two discrete levels of risk, it may also contribute to differences in replication across studies. These current findings broadly converge with the conclusion of Mata et al. (2011) that learning-related change in cognitive capacities may lead to poorer decision-making. However, the current findings suggest that seniors may still be characterized by risky decision-making even when learning abilities are comparable with younger subjects.

### Cognitive modeling suggests age-related alteration of reward sensitivity

Cognitive modeling is useful for revealing latent parameters that underlie complicated patterns of behavior, especially when some behaviors (pops) but not others (pumps, earnings) significantly differ between groups. Hierarchical Bayesian estimation revealed that group level performance differences were reflective of variance in a latent reward sensitivity parameter, and did not relate to a change in behavioral consistency. This dissociation between reward sensitivity and response variability processes is supported by a rat study that demonstrated how inactivation of different cortical structures (mPFC and OFC, respectively) selectively alters these two performance features (Jentsch et al., 2010). Critically, participants had a similar estimation of the task structure and probability of explosions, suggesting that apparent increases in reward sensitivity were not due to poorer learning of risky probabilities.

A larger reward sensitivity parameter will directly scale with an increased probability of inflating the balloon on each trial. While the ANOVA was non-significant for a group difference in pumps, it can be seen in Figure 2A that seniors had a larger number of pumps in the high risk condition compared to young participants [in fact, this difference was nearly statistically significant in the high risk condition: *t*(51) = 1.95, *p* = 0.056, but not in the low risk condition: *t* < 1]. Convergent with this trend, increasing age predicted riskier decision-making following successes (Figure 3). Behavioral findings were suggestive of riskier decision-making in the high risk condition, but the statistical evidence did not support a strong conclusion from performance differences. Cognitive modeling provided strong support for a determination of altered decision-making, revealing an increased sensitivity to reward in seniors during high risk trials. In sum, an increased sensitivity to reward in high risk trials led seniors to pump more often, leading to both greater pops and higher cash-out earnings on a smaller number of trials; these outcomes equated over trials to similar virtual earnings between age groups.

### Potential neural systems involved in altered decision-making in old age

There are tremendous individual differences in performance on reward-based decision-making tasks amongst seniors. These differences likely implicate a host of differentially contributing mechanisms that underlie altered decision-making. For example, while many seniors still perform comparably well to young participants on the Iowa Gambling Task, a much larger percentage of seniors perform poorly (Denburg et al., 2005). Poor-performing seniors also fail to show anticipatory skin conductance increases prior to advantageous choices (Denburg et al., 2006). Other investigations have described how seniors have reduced neural activity and diminished affective tone during loss anticipation (Wood et al., 2005; Samanez-Larkin et al., 2007). These previous findings suggest that a decoupled neuro-visceral response may contribute to an alteration in risky decision-making.

In line with other studies of risk and reward (Kuhnen and Knutson, 2005; Wrase et al., 2007), neuroimaging investigations have detailed how a wide range of frontal cortical and striatal areas are increasingly active in the BART task in conjunction with riskier decisions (Rao et al., 2008, 2010). Parkinson’s patients with impulse control disorders have lower resting blood flow and lower blood flow reactivity in the striatum during the BART (Rao et al., 2010). This specific type of Parkinsonian patient is also influenced by dopamine agonists to increase the number of pumps (Claassen et al., 2011). These imaging and pharmacological findings clearly implicate increased cortico-striatal activity with impulsive risk taking during the BART task. Both decreased neuro-visceral integration during risk and increased cortico-striatal reactivity to reward offer plausible hypotheses for age-related neural changes that could underlie the pattern of effects observed here.

## Conclusion

This investigation revealed that increased age was associated with altered behavioral performance reflective of greater reward sensitivity during high risk decisions. Age-related structural or functional change in neural systems underlying neuro-visceral integration and reward responsiveness are plausible candidates for this specific developmental change in decision quality.

## Conflict of Interest Statement

The authors declare that the research was conducted in the absence of any commercial or financial relationships that could be construed as a potential conflict of interest.
